# Pressure support ventilation with minimal sedation as the main ventilatory mode in critically ill patients with lung injury: effect on mortality and incidence of complications

**DOI:** 10.1186/cc12034

**Published:** 2013-03-19

**Authors:** A Gomez, A Leon, G Fernandez, G Montenegro, H Gomez

**Affiliations:** 1 Clinica Palermo, Bogota, Colombia; 2 University of Pittsburgh, PA, USA

## Introduction

The primary aim of this study is to assess the impact of pressure support ventilation (PSV) on the rate of pneumothorax and mortality in critically ill patients with lung injury. The secondary aim is to evaluate pressure-volume (P-V) relationships. Spontaneous modes of ventilation have been associated with lower rates of atelectasis, less muscle atrophy, better airflow distribution and importantly lower sedation requirements, which relates to lower mortality. Accordingly, we hypothesized that the use of PSV in patients with moderate/severe lung injury would have rates of pneumothorax and mortality within the standard of care. We further hypothesized that given its spontaneous nature, set pressures (PEEP and PS) but not tidal volume (Vt) would be related to airway pressures.

## Methods

All adult patients admitted to two surgical/medical ICUs subjected to invasive mechanical ventilation (MV) were enrolled. Patients were stratified by Lung Injury Score (LIS) in two groups: <2.5 (LISL); ≥2.5 (LISH). Exclusion criteria included pneumothorax on admission, use of other ventilatory strategies, and inability to trigger ventilation. Patients were ventilated with PSV, and treated only with *pro re nata *haldol, morphine and clozapine. Airway pressures and ventilatory mechanics were measured twice daily. Data are presented as mean ± 95% CI. *P > 0.05.

## Results

A total of 166 patients with mean age 55 (52 to 58) years, LIS of 2.27 (2.16 to 2.37) and MODS of 3.13 (2.9 to 3.3) were enrolled and stratified by LISL (65.8%) and LISH (34.2%). Mortality, and incidence of pneumothorax and atelectasis were 21% (14.9 to 28.2), 1.6% (0.8 to 3.1) and 6.4 (3.2 to 11.2). Duration of MV was 6 days (4.83 to 7.16). Pneumothorax in LISL and LISH was 1% (0 to 5.2) versus 2.4% (0.7 to 6.1)*, and mortality 11.4% (3.8 to 24.6) versus 25% (9.8 to 46.7)*. Mean parameters were: Vt 10.3 ml/kg (10.1 to 10.6); PEEP 10.6 (9.9 to 11); PS 16.6 (15.9 to 17.1); plateau pressure (Ppl) 25.7 (25.1 to 26.2). Ppl was >26.2 only in 2.5%. PEEP and PS (*P <*0.0001), and MODS were associated with Pplt, but not Vt* or LIS. Only lower Vt was associated with barotrauma (OR = 0.996, *P *= 0.02).

## Conclusion

We demonstrate that PSV in minimally sedated patients with severe lung injury is safe as it is associated with low incidence of barotrauma, atelectasis and mortality, and with Ppl and duration of MV within standard of care. We also demonstrate in PSV that P-V relationships may differ and that in this setting higher Vt may not be deleterious.

